# IABP versus Impella Support in Cardiogenic Shock: “In Silico” Study

**DOI:** 10.3390/jcdd10040140

**Published:** 2023-03-26

**Authors:** Beatrice De Lazzari, Massimo Capoccia, Roberto Badagliacca, Selim Bozkurt, Claudio De Lazzari

**Affiliations:** 1 Human Movement and Sport Sciences, “Foro Italico” University of Rome, 00147 Rome, Italy; beatrice.delazzari@gmail.com; 2 Northern General Hospital, Sheffield Teaching Hospitals NHS Foundation Trust, Sheffield S5 7AU, UK; capoccia@doctors.org.uk; 3 Department of Biomedical Engineering, University of Strathclyde, Glasgow G4 0NW, UK; 4 Department of Clinical, Internal Anesthesiology and Cardiovascular Sciences, “Sapienza” University of Rome, 00147 Rome, Italy; 5 School of Engineering, Ulster University—Belfast, United Kingdom of Great Britain and Northern Ireland, York Street, Belfast BT15 1 AP, UK; s.bozkurt1@ulster.ac.uk; 6 National Research Council, Institute of Clinical Physiology (IFC-CNR), 00185 Rome, Italy; claudio.delazzari@ifc.cnr.it; 7 Faculty of Medicine, Teaching University Geomedi, 0114 Tbilisi, Georgia

**Keywords:** IABP, Impella, cardiogenic shock, ventricular elastance, chronic heart failure, lumped parameter model, software simulation, cardiovascular modelling, CARDIOSIM^©^

## Abstract

Cardiogenic shock (CS) is part of a clinical syndrome consisting of acute left ventricular failure causing severe hypotension leading to inadequate organ and tissue perfusion. The most commonly used devices to support patients affected by CS are Intra-Aortic Balloon Pump (IABP), Impella 2.5 pump and Extracorporeal Membrane Oxygenation. The aim of this study is the comparison between Impella and IABP using CARDIOSIM^©^ software simulator of the cardiovascular system. The results of the simulations included baseline conditions from a virtual patient in CS followed by IABP assistance in synchronised mode with different driving and vacuum pressures. Subsequently, the same baseline conditions were supported by the Impella 2.5 with different rotational speeds. The percentage variation with respect to baseline conditions was calculated for haemodynamic and energetic variables during IABP and Impella assistance. The Impella pump driven with a rotational speed of 50,000 rpm increased the total flow by 4.36% with a reduction in left ventricular end-diastolic volume (LVEDV) by ≅15% to ≅30%. A reduction in left ventricular end systolic volume (LVESV) by ≅10% to ≅18% (≅12% to ≅33%) was observed with IABP (Impella) assistance. The simulation outcome suggests that assistance with the Impella device leads to higher reduction in LVESV, LVEDV, left ventricular external work and left atrial pressure-volume loop area compared to IABP support.

## 1. Introduction

Cardiogenic shock (CS) is part of a clinical syndrome consisting of acute left ventricular failure causing severe hypotension leading to inadequate organ and tissue perfusion. Recovery is observed if the underlying cause is reversible and appropriate treatment is started promptly. CS may become irreversible if treatment is delayed leading to severe tissue damage and death even if blood pressure is restored [1].

The most frequent cause of cardiogenic shock is heart failure (HF) secondary to acute myocardial infarction, although other conditions such as arrhythmias, valve rupture, pulmonary embolus, pericardial tamponade and acute myocarditis may lead to its development. CS is characterised by the following haemodynamic criteria: systolic aortic pressure (SAP < 90 mmHg), cardiac index (CI < 1.8 L/min/m^2^ without support or CI < 2.2 L/min/m^2^ with support), pulmonary capillary wedge pressure (PCWP > 15 mmHg), and elevated left ventricular end-diastolic pressure (LVEDP > 18 mmHg) [2]. 

Early pharmacological treatment may help avoid further worsening of the clinical picture and escalate to mechanical circulatory support. The most used drugs are positive inotropes (to increase the contractile force of the myocardium).

The most commonly used devices to support patients affected by CS are the following:

✓ Intra-aortic balloon pump (IABP), consisting of a balloon positioned in the descending thoracic aorta that inflates (diastole) and deflates (systole) leading to an increase in coronary perfusion and a reduction in afterload;
✓ Impella 2.5 [3], a coaxial pump that is retrogradely advanced in the aortic transvalvular position and works by aspirating blood from the left ventricle to expel it directly into the ascending aorta. This pump can deliver a flow of up to 2.5 L per minute;
✓ Extracorporeal membrane oxygenation (ECMO), which can simultaneously provide mechanical support for the heart and oxygenation of the lungs.

The correct choice of the device, the timing of the implant, the duration of the support and the prevention of any complications represent the key management points in patients requiring mechanical circulatory support (MCS). 

Percutaneous mechanical circulatory support has increased in recent years.

Although the IABP still remains a suitable choice, alternative devices have been introduced in clinical practice with particular reference to the Impella device and its use in an acute setting. Analysis of the available literature suggests that there is insufficient evidence to support superiority of IABP versus Impella when comparing survival rates [4] despite possible higher haemodynamic support offered by the Impella device [5], particularly when implanted before percutaneous revascularisation [6]. As the scientific evidence remains controversial, currently different centres follow local policy and experience in relation to decision-making and insertion/removal techniques.

The aim of this study is the comparison between Impella 2.5 and IABP using CARDIOSIM^©^ [7,8,9,10,11,12,13,14] software simulator of the cardiovascular system. Our study may contribute to fill the gap in the limited available data from other studies directly comparing Impella 2.5 with IABP.

For the purposes of this study, we reproduced the CS status of a virtual patient using an upgraded version of CARDIOSIM^©^, which has been developed in the “Cardiovascular Numerical/Hybrid Modelling Lab” of the Institute of Clinical Physiology (IFC-CNR) based in Rome. Subsequently, assistance with IABP and Impella 2.5 pump was simulated to evaluate the effects induced on haemodynamic and energetic variables. Two new modules reproducing the behaviour of IABP and Impella 2.5 were implemented in the CARDIOSIM^©^ platform to simulate the effects induced by the two devices in cooperation with the Faculty of Human Movement and Sport Sciences, “Foro Italico” University of Rome.

## 2. Materials and Methods

### 2.1. The Cardiovascular and Heart Numerical Models

**Figure 1:**
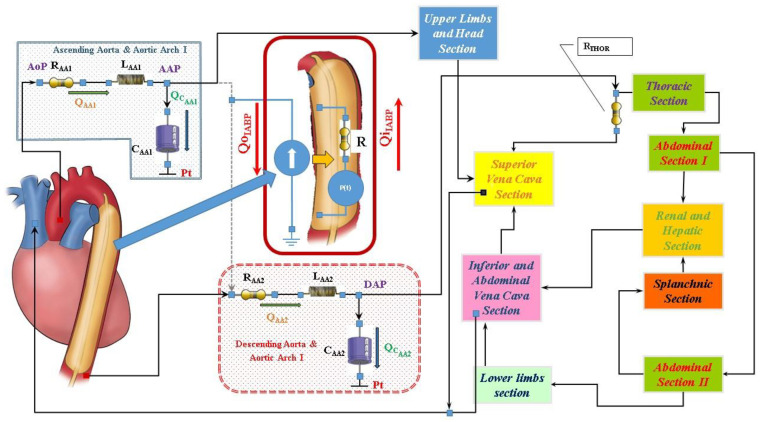
Schematic representation of the cardiovascular system assembled with the IABP. RLC elements in the ascending (descending) aorta and aortic arch compartment represent resistance, inductance and compliance, respectively. Pt is the intrathoracic pressure.

The numerical model of the cardiovascular system used to perform our simulations has been previously described [7,8,9,10,11]. The electric analogue of the cardiovascular network described in [12] consists of the following compartments (Figure 1): ascending and descending aorta with aortic arch, thoracic, upper limbs and head, superior and inferior vena cava, renal and hepatic, splanchnic, abdominal and lower limbs [12]. All the compartments are developed using lumped parameter (0-D) models. Both atrial and ventricular septa are interdependent, and they are modelled using the time-varying elastance approach [8]. Mitral, tricuspid, pulmonary and aortic valves are modelled using resistance and diode. A model with inverse resistance is used to simulate pulmonary and tricuspid regurgitation [7,9]. The numerical model of the coronary circulation assembled in this configuration of the cardiovascular system is presented in [13,14] (see supplementary materials).

### 2.2. Intra-Aortic Balloon Pump Numerical Model

Figure 1 shows the electric analogue of IABP inserted below the origin of the left subclavian artery and, therefore, placed after the ascending aorta and aortic arch compartment. The intra-aortic balloon pump is considered as a flow source Q_IABP_(t) in the following way [15,16,17,18,19]:

- the balloon inflates in diastole and the flow is positive;
- the balloon deflates in the following systole and the flow is negative.

**Figure 2:**
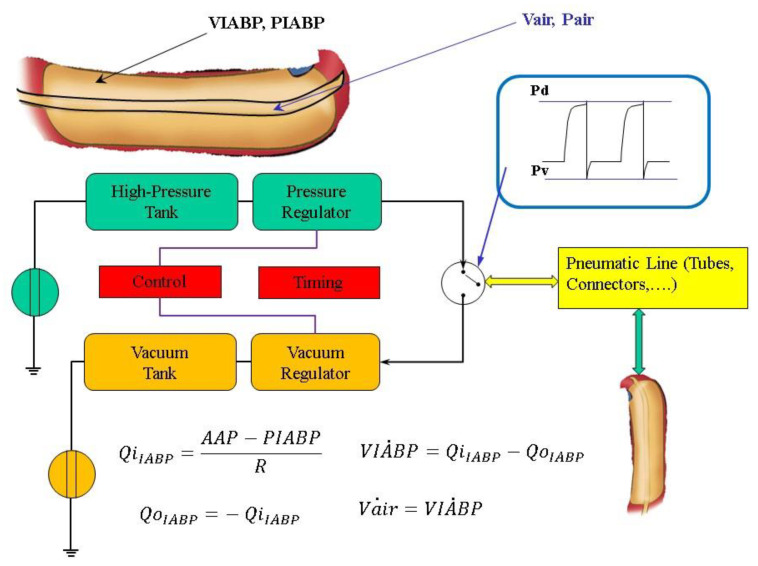
General layout of the driving unit of the IABP system. The air pressure waveform in the balloon is in the top right-hand corner. *Pd* and *Pv* are the driving and vacuum pressure, respectively. *Qi_IABP_* (*Qo_IABP_*) represents the input (output) flow source. $\dot{Vair}$ (*Pair*) is the volume (pressure) into the part of the balloon connected to the air tube; $\dot{VIABP}$ is the balloon volume, *Vmax* is the maximum extension volume of the balloon; $PIABP=\left(\left[\frac{Pair*760}{1000}\right]-760\right)$ is the balloon pressure; AAP is the ascending aorta pressure.

The flow source Q_IABP_(t) may be replaced by a pneumatic pressure source *P(t)*, representing the compressed gas reservoir, and by resistance (*R*) representing the total gas delivery resistance of the system. The pneumatic source *P(t)* has been modelled describing the ejection and the filling phase separately as follows:

- the air outflow from the high-pressure tank connected to the pressure source;
- the air outflow from the lower-pressure tank connected to the vacuum source (Figure 2).

IABP deflation is modelled by:

(1) $$\dot{Pair}=-\frac{1}{Vair^{-6}}\cdot \left[Kd\cdot Pair\cdot \left(\sqrt{E1\cdot \left[{\left(\frac{Pv}{Pair}\right)}^{E2}-{\left(\frac{Pv}{Pair}\right)}^{E3}\right]}\right)+Pair^{-6}\cdot \dot{Vair}\right]\;=-\frac{1}{Vair^{-6}}\cdot \left[Kd\cdot Pair\cdot \left(\sqrt{E1\cdot \left[{\left(\frac{Pv}{Pair}\right)}^{E2}-{\left(\frac{Pv}{Pair}\right)}^{E3}\right]}\right)+Pair^{-6}\cdot \left(Qi_{IABP}-Qo_{IABP}\right)\right]$$

IABP inflation is described by:

(2) $$\dot{Pair}=\frac{1}{Vair^{-6}}\cdot \left[Ks\cdot Pd\cdot \left(\sqrt{E1\cdot \left[{\left(\frac{Pair}{Pd}\right)}^{E2}-{\left(\frac{Pair}{Pd}\right)}^{E3}\right]}\right)-Pair^{-6}\cdot \dot{Vair}\right]\;=\frac{1}{Vair^{-6}}\cdot \left[Ks\cdot Pd\cdot \left(\sqrt{E1\cdot \left[{\left(\frac{Pair}{Pd}\right)}^{E2}-{\left(\frac{Pair}{Pd}\right)}^{E3}\right]}\right)-Pair^{-6}\cdot \left(Qi_{IABP}-Qo_{IABP}\right)\right]$$

where *Vair = Vt + Vmax-VIABP*, *Vt* is the drive tube volume *Vt* = 160 [mL], *Vmax* is the maximum balloon volume *Vmax* = 195 [mL], *E*1 = 3.5, *E*2 = 1.42857, *E*3 = 1.71428, *Ks* = 0.000799 and *Kd* = 0.00128. 

The module implemented in the new configuration of CARDIOSIM^©^ enables adjustment of the driving and vacuum pressures, the balloon volume and the timing of the IABP The simulator allows also synchronisation of the IABP timing with the QRS complex of the ECG signal or with the aortic pressure waveform. Weaning from IABP can be simulated by decreasing the balloon augmentation ratio from 1:1 to 1:2 or 1:4 or 1:8 (see supplementary materials).

### 2.3. Impella 2.5 Numerical Model

**Figure 3:**
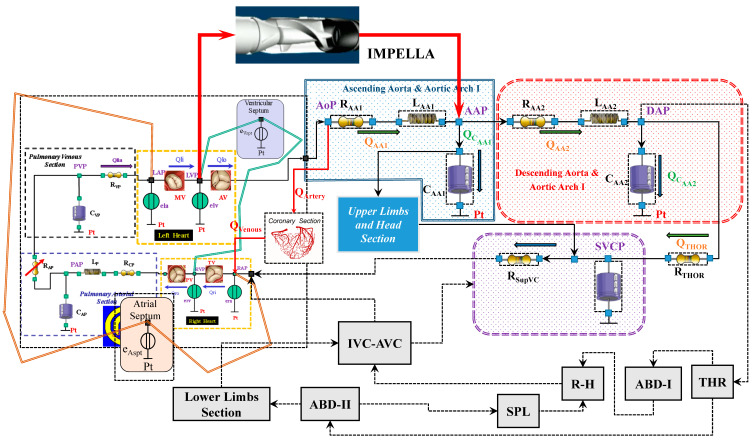
Schematic representation of the cardiovascular system assembled with the IMPELLA 2.5 pump.

Impella 2.5 is a catheter-based mechanical device designed to offer circulatory support through percutaneous insertion [3]. This pump is connected as left ventricular assist device (LVAD) across the aortic valve, generating blood flow in the ascending aorta with direct pressure and volume unloading. Figure 3 shows the schematic representation of the cardiovascular system assembled with the Impella 2.5 pump. 

The Impella flow (*F_IMP_*) obtained for different rotational speed is calculated using the following equation:

(3) $$F_{IMP}=K_{1}\cdot {\left(AAP-LVP\right)}^{4}+K_{2}\cdot {\left(AAP-LVP\right)}^{3}+K_{3}\cdot {\left(AAP-LVP\right)}^{2}+K_{4}\cdot \left(AAP-LVP\right)+K_{5}$$

The values of *K_i_*_(i=1,..,5)_ constants are listed in Table 1; LVP is the left ventricular pressure.

**Figure 4:**
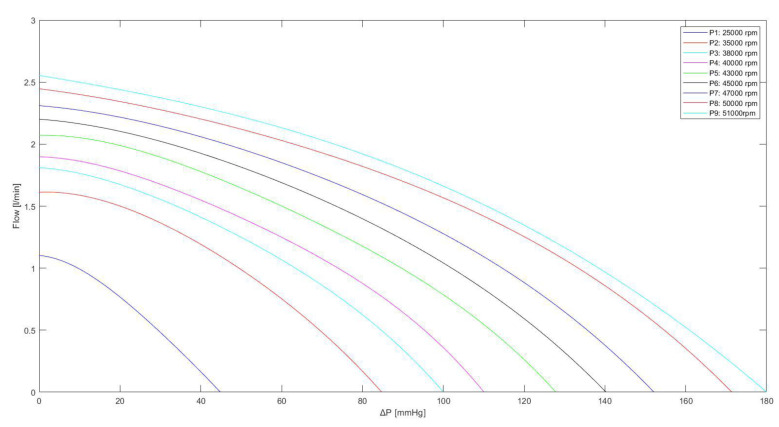
Relationship between the flow through the Impella 2.5 pump and the pressure difference for different rotational speeds. The curves were obtained using Equation (3), with the values listed in Table 1.

Equation (3) is used to derive the curves in Figure 4, which are in good agreement with the experimental data measured during the functioning of Impella 2.5 for different pump speeds ranging from 25,000 to 51,000 rpm [3,20].

### 2.4. Simulation Protocol

The benchmark for our simulations consisted of a virtual patient in cardiogenic shock whose baseline conditions included a systolic aortic pressure SAP = 79.3 mmHg, heart rate HR = 70 beat/min, mean left atrial pressure LAP = 21.3 mmHg, mean pulmonary arterial pressure PAP = 25.7 mmHg, LVEDP = 24 mmHg, cardiac output CO = 3.29 L/min, mean coronary blood flow CBF = 100.5 mL/min, cardiac index CI = 1.73 L/min/m^2^, LVEDV = 149.9 mL, LVESV = 103.0 mL, left ventricular ejection fraction EF% = 31.3 and left (right) ventricular arterial coupling E_a_/E_es_ = 1.71 (E_es_/E_a_ = 1.43).

IABP support was initiated in synchronised mode at baseline conditions with a delay of 220 ms from the start of ventricular diastole for balloon inflation and timing of deflation before the next systole. IABP assist ratio was 1:1 (one inflation per cardiac cycle), driving pressure was set to 260 mmHg with vacuum pressure at −10, zero and +10 mmHg, respectively. The percentage variation with respect to baseline conditions was calculated during IABP assistance for the following parameters: left ventricular output (or cardiac output CO), total flow (CO + Impella flow), cerebral and renal flow, left ventricular external work (LVEW), left ventricular ejection fraction (LVEF), systolic aortic pressure (SAP), end-diastolic aortic pressure (DAP), mean aortic pressure (AoP), LAP, RAP, PAP, CBF, left ventricular end-diastolic (end-systolic) volume LVEDV (LVESV) and left ventricular-arterial coupling (E_a_/E_es_).

Subsequently, LVAD assistance with Impella 2.5 was initiated with different rotational speeds (35,000, 45,000 and 50,000 rpm). The percentage variation with respect to baseline conditions was calculated for the above parameters.

## 3. Results

**Figure 5:**
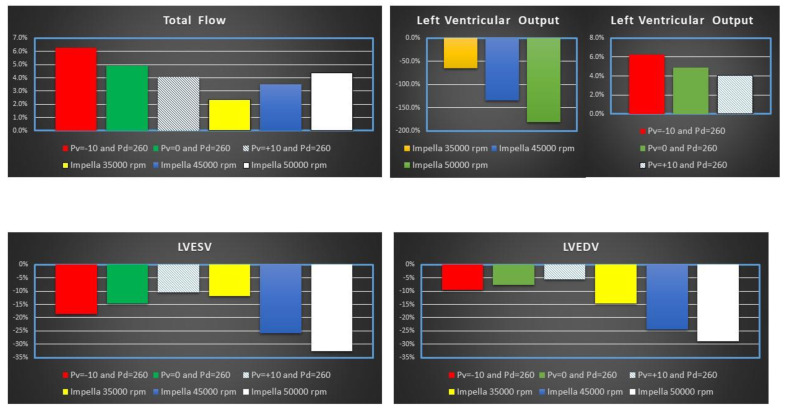
Percentage change calculated in comparison to baseline conditions for total flow, left ventricular output (LVO), LVEDV and LVESV when IABP or Impella 2.5 (LVAD) support were simulated. The simulation settings included LVAD rotational speed at 35,000, 45,000 and 50,000 rpm and IABP support with *Pv* = −10, *Pv* = 0 and *Pv* = +10 mmHg, and *Pd* = 260 mmHg. LVO (or CO) is the Total Flow under IABP support. The sum of CO and LVAD flow gives the total flow under Impella 2.5 support.

Figure 5 shows the percentage change of total flow (top left panel), LVOF (top right panel) LVESV (bottom left panel) and LVEDV (bottom right panel) calculated in comparison to baseline conditions for IABP and Impella 2.5 (LVAD) support. The simulation settings included LVAD rotational speed at 35,000, 45,000 and 50,000 rpm and IABP support with *Pv* = −10, *Pv* = 0 and *Pv* = +10 mmHg, and *Pd* = 260 mmHg. 

**Figure 6:**
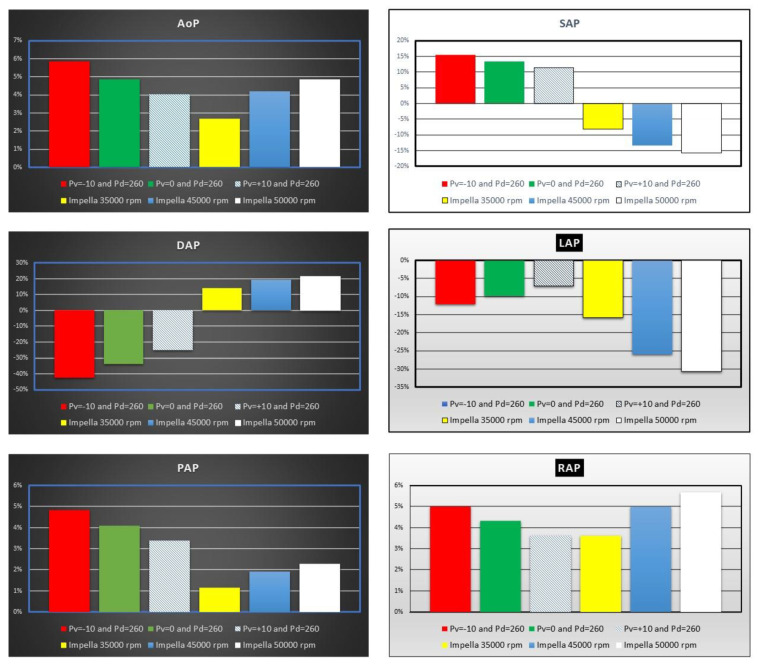
Percentage change calculated in comparison to baseline conditions for mean aortic pressure (AoP), systolic and end-diastolic aortic pressure (SAP and DAP), mean left atrial pressure (LAP), mean pulmonary arterial pressure (PAP) and mean right atrial pressure (RAP) when IABP or Impella 2.5 support were simulated. The simulation settings included LVAD rotational speed at 35,000, 45,000 and 50,000 rpm and IABP with *Pv* = −10, *Pv* = 0 and *Pv* = +10 mmHg, and *Pd* = 260 mmHg.

Impella 2.5 support reduced LVO (or CO) by ≅180% (from 3.29 to 1.17 L/min) when the rotational speed was set to 50,000 rpm (top left panel in Figure 4). Consequently, the pump increased the total flow by 4.36% (from 3.29 to 3.44 L/min) with a pump flow of 2.27 L/min. The top left panel in Figure 5 shows that IABP assistance increased LVO from ≅4% (*Pv* = +10 mmHg) to ≅6.3% (*Pv* = −10 mmHg). Both Impella 2.5 and IABP reduced LVESV (bottom left panel) and LVEDV (bottom right panel). A reduction in LVEDV by ≅15% to ≅30% was observed on LVAD support. Volume unloading on IABP was only 5–10%. The bottom left panel in Figure 4 shows that a reduction in LVESV by ≅10% to ≅18% (≅12% to ≅33%) was observed on IABP (Impella) assistance [21]. Figure 6 shows the effects induced by IABP and Impella assistance on aortic blood pressure and left atrial pressure (LAP).

The top left panel shows an increase in mean aortic pressure (AoP) by 4% to ≅6% when IABP was activated. The simulation settings based on different rotational speed for Impella 2.5 pump show an increase in AoP by ≅2.5% to ≅5% in line with current published literature [4,22,23,24]. SAP decreased by ≅15% compared to baseline conditions when the LVAD rotational speed was set to 50,000 rpm (top right panel in Figure 6), but increased up to ≅15% when driving and vacuum IABP pressures were set to 260 and −10 mmHg, respectively. In contrast, DAP increased up to ≅22% when the Impella rotational speed was set to 50,000 rpm (middle left panel), whilst it decreased by ≅43% when driving and vacuum IABP pressures were set to 260 and −10 mmHg, respectively. The middle right panel (Figure 6) shows that IABP support (*Pv* = −10 and *Pd* = 260 mmHg) reduced LAP by ≅13% whilst Impella 2.5 assistance increased it by more than ≅30%. Mean pulmonary arterial (bottom left panel) and right atrial (bottom right panel) pressures showed similar percentage increase on IABP support. LVAD assistance increased mean PAP (RAP) up to ≅2.3% (≅5.7%) when the rotational speed was set to 50,000 rpm. 

**Figure 7:**
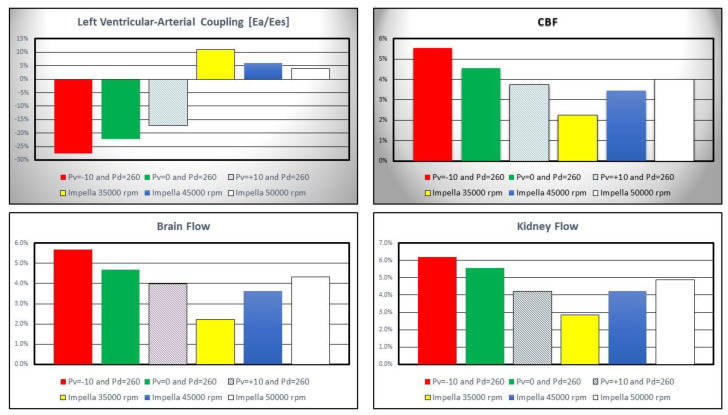
Percentage change calculated in comparison to baseline conditions for left ventricular-arterial coupling, coronary, cerebral and renal blood flow when IABP or LVAD support were simulated. The simulation settings included Impella 2.5 rotational speed at 35,000, 45,000 and 50,000 rpm and IABP support with *Pv* = −10, *Pv* = 0 and *Pv* = +10 mmHg, and *Pd* = 260 mmHg. *E_a_* is the arterial elastance and *E_es_* is the left ventricular elastance.

Figure 7 shows the percentage change in left ventricular-arterial coupling and coronary, cerebral and renal blood flow calculated in comparison to baseline conditions for IABP and Impella 2.5 assistance. 

IABP assistance reduced *E_a_*/*E_es_* by more than 25% when *Pv* = −10 and *Pd* = 260 mmHg whilst Impella pump increased left ventricular–arterial coupling although inversely related to pump rotational speed (top left panel). Coronary, cerebral and renal blood flow increased with both Impella and IABP support (top right and bottom left and right panel in Figure 7) [22,25].

**Figure 8:**
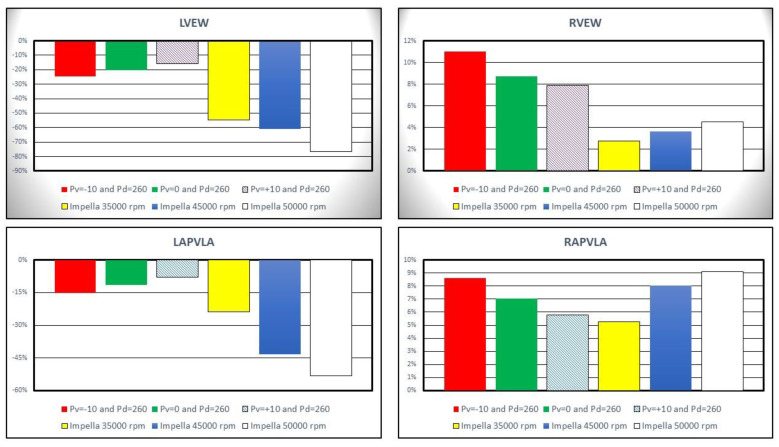
Percentage change calculated in comparison to baseline conditions for left ventricular external work (**top left panel**), right ventricular external work (**top right panel**) and left and right atrial pressure–volume loop area ((**bottom left panel**) and (**bottom right panel**)). The above values were obtained when the Impella rotational speed was set to 35,000, 45,000 and 50,000 rpm. During the simulations with IABP support the driving and vacuum pressures were set to *Pd* = 260 mmHg and to *Pv* = −10, *Pv* = 0 and *Pv* = +10 mmHg, respectively.

Left ventricular external work (LVEW) decreased by more than 20% compared to baseline conditions on IABP assistance with *Pd* = 260 mmHg and *Pv* = −10 mmHg (top left panel in Figure 8). Impella 2.5 support reduced LVEW by more than 75% (55%) at 50,000 (35,000) rpm. Left ventricular pressure-volume area (LPVA) decreased by ≅33.7% at 35,000 rpm and by ≅65.9% at 50,000 rpm [26,27]. LVPA is an index of myocardial oxygen consumption; therefore, increased pump rotational speed was related to a decrease in myocardial oxygen consumption. The top right panel in Figure 8 shows that the percentage change in right ventricular external work (RVEW) is the highest when IABP support is turned on. The percentage reduction in left atrial pressure–volume loop area (LAPVLA) is negligible under IABP support whilst a percentage variation in LAPVLA ranging between ≅24% (35,000 rpm) and ≅53.3% (50,000 rpm) is observed during Impella assistance (bottom left panel). Finally, the bottom left panel in Figure 8 shows that right atrial pressure–volume loop area (RAPVLA) is not significantly affected by the two devices.

**Figure 9:**
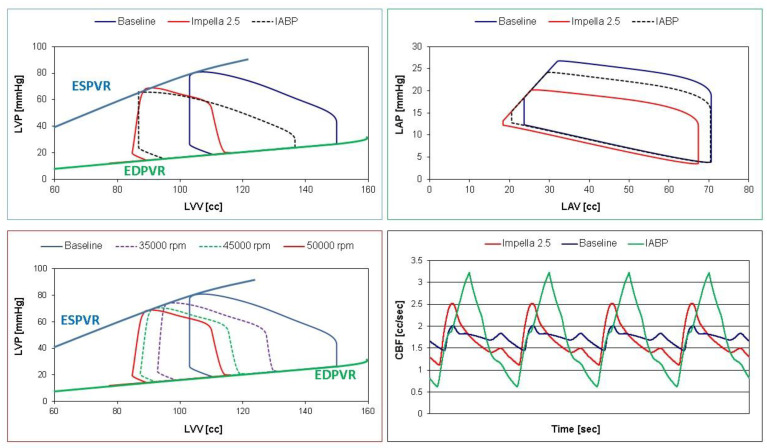
Left ventricular pressure–volume loops (**top and bottom left panels**) and coronary blood flow waveforms (**bottom right panel**) obtained storing data in Excel file and subsequently processed with Excel software. The (**top left (right) panel**) shows the left ventricular (atrial) pressure–volume loops obtained in baseline (blue continuous lines) and assisted conditions with IABP (*Pd* = 260 and *Pv* = −10 mmHg—dashed black lines) and Impella 2.5 (red lines) at 50,000 rpm. The (**bottom left panel**) shows the left ventricular pressure–volume loops obtained in baseline (blue continuous lines) and assisted conditions with LVAD rotational speed at 35,000, 45,000 and 50,000 rpm.

Simulation data were stored in Excel file and analysed with Excel software (Figure 9) to plot pressure–volume loops and coronary blood flow waveforms. 

The top left (right) panel in Figure 9 shows the left ventricular (atrial) pressure–volume loops obtained in baseline (blue line) and assisted conditions with Impella 2.5 (red line) and IABP (dashed black line). Both devices reduced LVEDV, LVESV (top left panel) and left atrial end-systolic and end-diastolic volume (top right panel). The bottom left panel (Figure 9) shows the different left ventricular pressure–volume loops in baseline (blue line) and assisted conditions with LVAD rotational speed at 35,000 (lilac dashed line), 45,000 (green dashed line) and 50,000 rpm (red line). Finally, the coronary blood flow waveforms (bottom right panel) in baseline (blue line) and assisted conditions with LVAD (red line) and IABP (green line) have been developed using Excel software.

**Figure 10:**
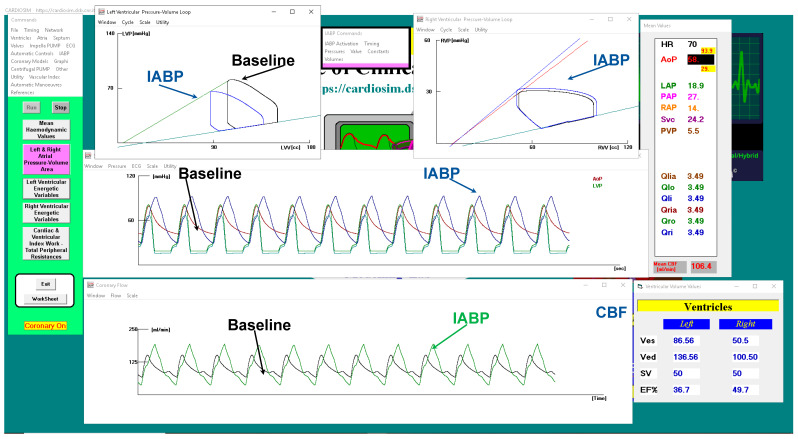
CARDIOSIM^©^ screenshot showing baseline and assisted conditions with IABP. Proceeding from the top left to the right and then down, the left (top left side) and right (top right side) ventricular pressure–volume loops for baseline and IABP support are observed. The left ventricular and aortic pressure waveforms for baseline (red line) and IABP assistance (blue line) are plotted in the middle window. The bottom window shows the coronary blood flow waveforms.

Figure 10 shows a screenshot of CARDIOSIM^©^ software simulator outlining baseline conditions and IABP support.

The driving and vacuum pressures were set to 260 mmHg and −10 mmHg, respectively, during IABP support. The green coronary blood flow waveforms are plotted in the bottom window (Figure 10) whilst the black line is the CBF in baseline conditions.

## 4. Discussion

The intra-aortic balloon pump has been widely used as first-line circulatory support device since its first introduction in clinical practice. Despite its proven effects, there has been controversy about its role in cardiogenic shock following the questionable outcome of the SHOCK trial [28,29,30]. Alternatives, such as the Impella device, have been proposed. This study has mainly addressed the performance of each device in terms of their strengths and weaknesses and determined their potential in the context of a virtual patient in cardiogenic shock. The outcome of IABP and Impella 2.5 support was compared with the use of numerical models describing blood flow rates and pressures in the cardiovascular system and IABP and Impella 2.5 features. Cardiogenic shock was simulated by tuning the parameters in the cardiovascular system model and the same settings were used to simulate the outcome of pump support. In addition, three different operating conditions were used in both IABP (*Pv* = −10 mmHg, 0 mmHg and 10 mmHg) and Impella 2.5 (35,000 rpm, 45,000 rpm and 50,000 rpm) to evaluate haemodynamic variables on pump support. The simulation results show that Impella 2.5 provided better left ventricular unloading than IABP as the decrease in left ventricular end-systolic and end-diastolic volumes were relatively high at 45,000 rpm and 50,000 rpm operating speeds (Figure 5). The simulation results are consistent with the clinical findings for left ventricular unloading [31]. 

Although both devices increased the mean aortic pressure with varying degree at different settings, the increase in the mean aortic pressure was higher on IABP support. IABP increased the systolic aortic pressure and decreased end-diastolic aortic pressure whereas Impella 2.5 decreased the systolic aortic pressure and increased end-diastolic aortic pressure. Therefore, aortic pulse pressure was higher on IABP support. Impella 2.5 decreased the aortic pulse pressure due to continuous operating speed and unloading of the left ventricle. IABP on the other hand was synchronised with the left ventricle and provided an increased aortic pulse pressure (Figure 6). Again, the simulation results confirm the clinical findings [31]. Impella 2.5 support reduced the mean left atrial pressure more than IABP at all operating speeds. Impella 2.5 support is more beneficial in reducing left atrial pressure.

Left ventricular–arterial coupling is ≅1 in healthy conditions [32], whereas it increases with reduced left ventricular end-systolic elastance during cardiogenic shock [33]. The baseline left ventricular–arterial coupling was 1.71 in the simulations confirming the clinical data. Coronary, cerebral and renal blood flow rates increased on both IABP and Impella 2.5 support. This was achieved by decreasing vacuum pressure for IABP or increasing operating speed for Impella 2.5. However, IABP support at *Pv* = −10 mmHg and *Pv* = 0 mmHg vacuum pressures increased the blood flow rates in these sections better than Impella 2.5 support (Figure 7) which may be interpreted as IABP being more beneficial to improve organ perfusion.

Impella 2.5 decreased left ventricular external work in a more remarkable manner compared to IABP (Figure 8). Therefore, Impella 2.5 seems to reduce myocardial oxygen consumption more effectively compared to IABP. A similar trend in the change of left atrial pressure–volume loop area occurs under the support of both IABP and Impella 2.5 devices. On the other hand, Impella 2.5 support increases right ventricular external work remarkably less than IABP at all simulated operating speeds (Figure 8). 

Each device has also been used combined with V-A ECMO [34,35]. A systematic review and meta-analysis of the combined use of V-A ECMO and IABP in cardiogenic shock has shown reduced in-hospital mortality without increased rate of complications [36]. A combined use of IABP and Impella has been proposed as a potentially superior approach for refractory cardiogenic shock [37,38] based on initial experimental evidence of favourable haemodynamics following combined support with the IABP and Impella p9 device in a sheep model of acute myocardial infarction [26]. Another porcine model of acute myocardial infarction has shown that LV unloading with Impella CP decreases LV end-diastolic wall stress and increases microvascular perfusion of the infarcted area [22]. A retrospective review of 128 patients undergoing V-A ECMO or Impella support because of refractory cardiogenic shock after acute MI showed significant reduction in adjusted 30-day mortality following V-A ECMO support. A higher rate of MCS escalation was observed in patients undergoing Impella device support [2]. A review of 6290 patients sustaining acute myocardial infarction complicated by cardiogenic shock and requiring percutaneous coronary intervention (PCI) showed better outcome for patients receiving Impella support compared to those undergoing V-A ECMO insertion [39]. An experimental model of porcine acute myocardial infarction has been used to compare peripheral V-A ECMO with Impella CP based on an open-labelled randomised setting. Impella CP resulted in a more effective volume unloading of the left ventricle. Both devices reduced myocardial oxygen consumption significantly. Impella CP shifted the pressure–volume loop to the left with a decreased pressure–volume–area (PVA) whilst V-A ECMO increased PVA and decreased heart rate [40]. 

The outcome of our simulations does confirm the more efficient volume unloading and reduction in myocardial oxygen consumption generated by Impella 2.5. Nevertheless, organ perfusion in terms of coronary, cerebral and renal blood flow is better addressed by IABP assistance. Our study has not directly evaluated the combined use of the two devices, which may be considered a limitation. Nevertheless, the overall analysis of data suggests that a combined use of IABP and Impella 2.5 may compensate the shortcomings of each device alone and potentially lead to a better level of support. The importance of the context remains to be taken into account considering that IABP availability is higher given its ease of use and patient transfer to a cath lab is not necessarily required: device insertion can be performed at the bedside in intensive care unit or in theatre with or without trans-oesophageal echocardiography guidance. Finally, our results may help with the implementation of a more patient-specific approach in terms of treatment optimisation and possibly outcome prediction with a view to identify those patients who may benefit the most.

## 5. Conclusions

The present study suggests that assistance with the Impella 2.5 device leads to significant unloading of the left ventricle with greater reduction in LAP, LVESV, LVEDV, left ventricular external work and left atrial pressure–volume loop area compared to IABP support. Notably, the level of improvement driven by IABP and Impella 2.5 is strongly dependent on the pathological haemodynamic scenario simulated.

## Figures and Tables

**Table 1:** Numerical model parameters for Impella 2.5.

Pump Rotational Speed (rpm)	K_1_	K_2_	K_3_	K_4_	K_5_
25,000	−1.157·10^−7^	1.622·10^−5^	−0.0009846	−0.002613	1.102
35,000	−2.065·10^−8^	3.849·10^−6^	−0.0004192	0.001435	1.612
38,000	−1.668·10^−8^	2.976·10^−6^	−0.0002915	0.002004	1.812
40,000	−1.497·10^−8^	3.849·10^−6^	−0.000417	0.0005987	1.898
43,000	−1.084·10^−8^	2.59·10^−6^	−0.0002857	0.0006554	2.071
45,000	−4.085·10^−9^	9.128·10^−7^	−0.0001425	−0.002385	2.201
47,000	−3.011·10^−9^	6.504·10^−7^	−0.0001116	−0.0026555	2.31
50,000	−1.742·10^−9^	3.015·10^−7^	−6.007·10^−5^	−0.004055	2.446
51,000	−1.845·10^−10^	−2.204·10^−7^	−1.528·10^−5^	−0.000537	2.554

## Data Availability

Not applicable.

## Supplementary Materials

The following supporting information can be downloaded at https://cardiosim.dsb.cnr.it/ (accessed on 6 November 2022).
